# Editorial: The Different Faces of Sickness

**DOI:** 10.3389/fpsyt.2021.735337

**Published:** 2021-08-31

**Authors:** Lena Rademacher, Julie Lasselin, Bianka Karshikoff, Jennifer E. Hundt, Harald Engler, Tanja Lange

**Affiliations:** ^1^Social Neuroscience Lab at the Translational Psychiatry Unit (TPU), Department of Psychiatry and Psychotherapy, University of Lübeck, Lübeck, Germany; ^2^Center of Brain, Behavior and Metabolism (CBBM), University of Lübeck, Lübeck, Germany; ^3^Department of Psychology, Stress Research Institute, Stockholm University, Stockholm, Sweden; ^4^Department of Clinical Neuroscience, Karolinska Institutet, Stockholm, Sweden; ^5^Department of Social Studies, University of Stavanger, Stavanger, Norway; ^6^Lübeck Institute of Experimental Dermatology (LIED), University of Lübeck, Lübeck, Germany; ^7^Institute of Medical Psychology and Behavioral Immunobiology, Center for Translational Neuro- and Behavioral Sciences, University Hospital Essen, University of Duisburg-Essen, Essen, Germany; ^8^Department of Rheumatology and Clinical Immunology, University of Lübeck, Lübeck, Germany

**Keywords:** sickness behavior, social behavior, psychoneuroimmunology, COVID-19, infection, depression, pain

Sickness not only includes symptoms that classically define an infection (e.g., fever, nausea, headache), but also comes along with profound neurobehavioral consequences for the infected individual. These include anhedonia, anorexia, pain, lethargy, fatigue, sleepiness, and social withdrawal, and are collectively called “sickness behavior” (1). Sickness behavior in the infected individual is triggered by mediators of the activated immune system that signal to the brain, thus linking the immunological (inflammatory) response with the psychological (behavioral) response to a pathogen. These inflammatory mediators include cytokines (e.g., interleukins, IL; tumor necrosis factor, TNF) that can be assessed in the circulation in addition to clinical markers of inflammation (e.g., C-reactive protein, CRP). Inflammation and sickness behavior are paralleled by neuroendocrine changes including activation of the autonomic nervous system and the hypothalamus-pituitary-adrenal (HPA) axis (1), which are both critical for the feedback regulation of the immune response.

Sickness also has consequences for the social environment. Unaffected partners, peers, and relatives of a sick person will adapt behaviorally by expressing e.g., fear of infection, and avoiding or helping behaviors - disease-control strategies that can be observed in animals as well (2, 3). However, the consequences can also extend beyond the immediate social environment, as the COVID-19 pandemic has shown: worldwide efforts to prevent disease by monitoring and restricting social interactions ultimately led to governmental actions that markedly affected the freedom and social life of individuals. The aim of this Research Topic was to bring together original research articles, reviews, and opinion articles that shed light on the interplay between the immune system, sickness (behavior) and social as well as psychological consequences. We here summarize ten accepted manuscripts that cover a wide range of these topics (see Figure 1).

**Figure 1:**
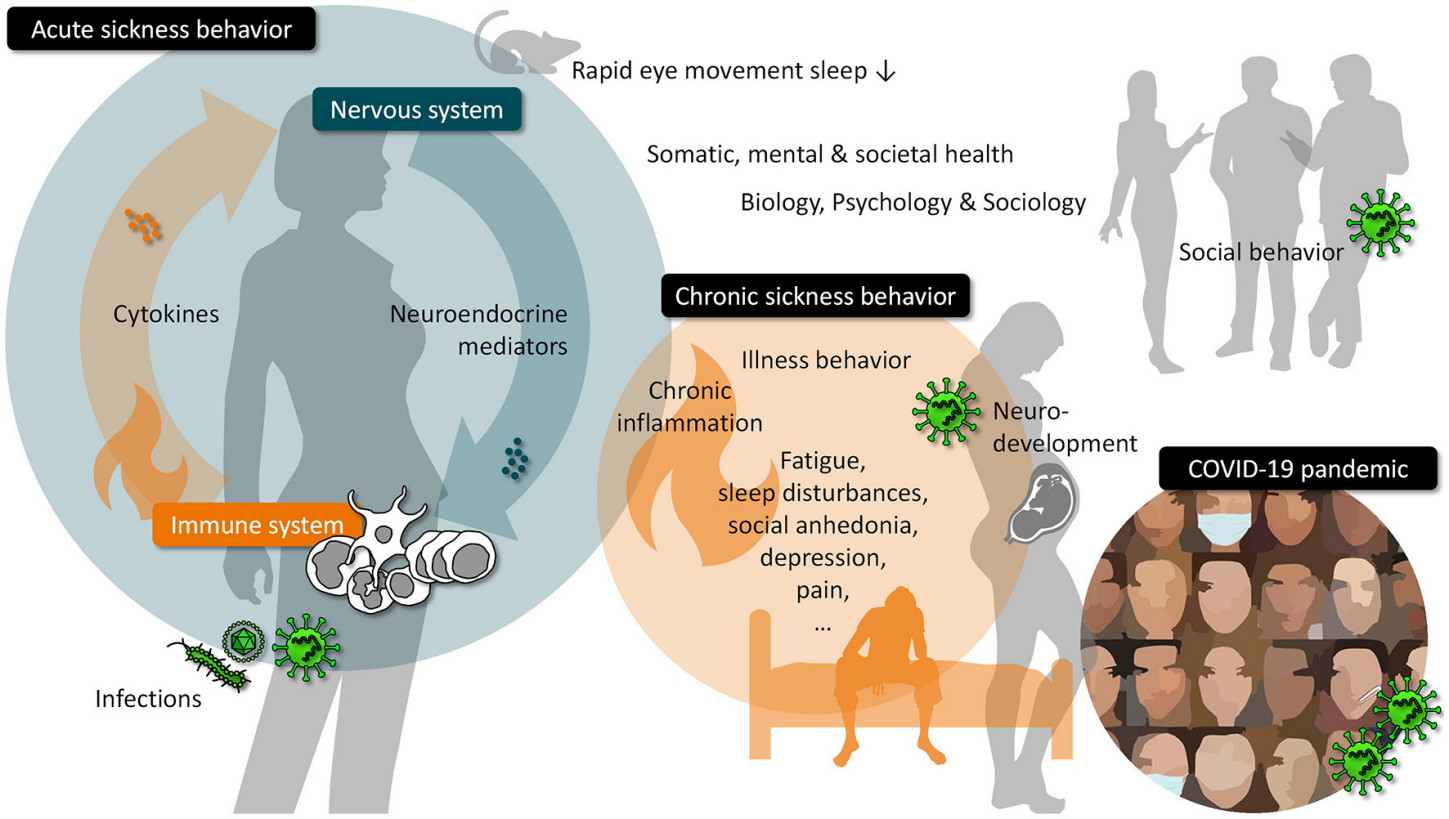
Overview on topics related to the interplay between the immune system, sickness (behavior) and social as well as psychological consequences.

Parts of the Research Topic address the concept of sickness behavior and provide insights into its historical development and the underlying neuro-immune mediators. Konsman discusses the concepts of sickness, disease, and illness, and what is considered to be “sickness behavior” and “illness behavior” across the fields of Biology, Psychology, and Sociology. Although these concepts have evolved differently between the fields, due to different traditions of research, many aspects are similar and compatible. This calls for an interdisciplinary approach to deepen our understanding of sickness behavior. Taking a historical perspective on fever and sickness behavior research, Kelley and Kent highlight the importance of concepts that link immunology and systemic physiology. They discuss immune to brain signaling pathways such as cytokines and central nervous responses to immune signals such as activation of the HPA-axis. One important neuronal target region of immune signals is the hypothalamus, which regulates fever, stress systems, sleep-wake behavior, food intake, and social behavior. Using fiber photometry in mice, Borniger and de Lecea demonstrate that activity of GABAergic neurons in the lateral hypothalamus is highest during rapid eye movement (REM) sleep. Immune activation by injection of lipopolysaccharide acutely suppressed the activity of these neurons and eliminated REM sleep behavior.

All aspects of sickness behavior in response to acute infection, including changes in sleep, are seen as adaptive, as they presumably serve immune defense and recovery, e.g., by saving energy that can be re-allocated to the fighting immune system (4). In chronic conditions, however, long-lasting sickness behavior can become maladaptive and contribute to disease symptoms. In recent years, many studies have suggested a causal link between ongoing inflammation and impaired mental health (1). Apart from depressive symptoms, this may also hold true for other aspects of sickness behavior such as pain and fatigue. However, in populations with low-grade to normal cytokine levels, or subclinical distress, the relationship between cytokine levels and clinical features may be complex. Straka et al. use a community sample of 2,077 individuals to investigate the effect of age on the, at this point, fairly established relationship between inflammatory markers and depressive states. They show that while a positive relationship was seen between the inflammatory markers IL-6 and CRP and somatic complaints, a negative relationship between these markers and anhedonia appeared. Most importantly, these effects were stronger with increasing age. Karshikoff et al. investigate the relationship of inflammatory markers and clinical features in 261 patients with pelvic pain. This population has similar inflammatory levels as the healthy controls, and a negative correlation was seen between the pro-inflammatory cytokine IL-8 and the widespreadness of pain, while a positive relationship was seen for the regulatory cytokine granulocyte-macrophage colony-stimulating factor. This study also suggests that fatigue is more strongly related to cytokine levels in the chronic pain group, than pain intensity or depressive symptoms. These findings suggest that inflammatory mechanisms related to pain and depression are complex and multifactorial. Munk et al. build on this notion and propose to apply the Cognitive Activation Theory of Stress (CATS) to chronic post-surgical pain after breast cancer surgery. CATS is a psychobiological theoretical framework bringing expectancies at the center of the stress response and health outcomes. Munk et al. argue that coping (“the acquired expectancy that most or all responses to a situation will lead to a positive outcome”), helplessness (“the acquired expectancy of one's actions having no impact on the outcome of an aversive event”), and hopelessness (“the expectancy of most or all responses leading to negative outcomes”) can modulate the physiological stress response to the post-surgical pain in women with breast cancer. A sustained stress response can in turn lead to chronic pain, through mechanisms such as chronic inflammation, central sensitization, and cortisol dysfunction, and could be treated by targeting outcome expectancies, for instance using Acceptance and Commitment Therapy or hypnosis. These examples of clinical conditions emphasize the importance of understanding acute, physiological, adaptive brain-immune interactions, and their chronic, pathological, maladaptive dysregulation in patients.

In addition to ongoing inflammation, maternal infection during pregnancy has also been identified as a risk factor for mental disorders with neurodevelopmental etiology. When occurring during vulnerable phases of fetal brain development, infection of the mother can change the offspring's neurodevelopmental trajectories and can increase its risk to develop a severe mental illness such as schizophrenia, autism spectrum disorder, and bipolar disorder later in life (5). Against this background, Reyes-Lagos et al. discuss in their perspective article the potential implications and long-term consequences of the COVID-19 pandemic on mental health, and illustrate why there is an urgent need for longitudinal studies in affected pregnant women and their offspring. Given the important role of the cholinergic anti-inflammatory pathway for immune homeostasis they propose to analyze this pathway during gestational infection with SARS-CoV-2, and to explore vagus nerve stimulation or the use of cholinergic agonists as therapeutic options for dampening virus-induced maternal and fetal inflammation.

One objective of this Research Topic was to consider the interplay between immune activation and social behavior. In their review, Smith and Bilbo summarize literature on the bi-directional relationship between immune functions and social life. They describe how infection and inflammation alter social behavior and how, on the other hand, social experiences can influence the immune system. Smith and Bilbo additionally give an outlook on how these findings might be relevant in the context of the COVID-19 pandemic. Two other manuscripts focus specifically on the social and psychological consequences of the COVID-19 pandemic. In their review, Saladino et al. describe the impact of the pandemic on the well-being and mental health of children, young adults, as well as health care workers. Furthermore, the authors discuss the consequences for interpersonal relationships and interactions and consider the potential role of online psychological support and psychotherapy. Stierand et al. investigate how the appraisal of situations involving close or distant contact with other people changed from before to during the first wave of the COVID-19 pandemic in Germany. They report that both the risk associated with a particular situation and the individual's general perception of current infection risk are related to changes in comfort in social situations. These articles show the far-reaching consequences of a virus pandemic and the associated measures for the psyche and for social interactions.

In conclusion, our Research Topic brought together scientists from different disciplines, reporting basic and clinical research in animals and humans to delineate brain-immune interactions in systemic physiology and in clinical conditions, and to understand the immunological mechanisms of mental illness, pain, fatigue, and social anhedonia across the lifespan. The COVID-19 pandemic has featured the relevance of brain-immune interactions for somatic, mental, and societal health, and the need for comprehensive, longitudinal studies that monitor immunological, neurobehavioral, and psychological parameters in parallel. Only with such an interdisciplinary approach we might one day understand the transition from acute, adaptive to chronic, maladaptive conditions and thus the complexity of sickness behavior.

## Author Contributions

All authors have contributed to writing the manuscript.

## Conflict of Interest

The authors declare that the research was conducted in the absence of any commercial or financial relationships that could be construed as a potential conflict of interest.

## Publisher's Note

All claims expressed in this article are solely those of the authors and do not necessarily represent those of their affiliated organizations, or those of the publisher, the editors and the reviewers. Any product that may be evaluated in this article, or claim that may be made by its manufacturer, is not guaranteed or endorsed by the publisher.
